# A lifestyle score in childhood and adolescence was positively associated with subsequently measured fluid intelligence in the DONALD cohort study

**DOI:** 10.1007/s00394-022-02921-z

**Published:** 2022-06-15

**Authors:** Maike Elena Schnermann, Christina-Alexandra Schulz, Christine Ludwig, Ute Alexy, Ute Nöthlings

**Affiliations:** Department of Nutrition and Food Sciences, Institute of Nutrition and Food Sciences, Nutritional Epidemiology, University of Bonn, Friedrich-Hirzebruch-Allee 7, 53115 Bonn, Germany

**Keywords:** Combined lifestyle, Lifestyle score, Fluid intelligence, Cognitive ability, Children, Adolescents

## Abstract

**Purpose:**

Lifestyle scores which combine single factors such as diet, activity, or sleep duration showed associations with cognitive decline in adults. However, the role of a favourable lifestyle in younger age and the build-up of cognitive reserve is less clear, which is why we investigated longitudinal associations between a lifestyle score in childhood and adolescence and fluid intelligence obtained on average 6 years later.

**Methods:**

In the DONALD cohort, a lifestyle score of 0 to 4 points including healthy diet and duration of moderate-to-vigorous physical activity, sedentary behaviour and sleep was repeatedly assessed in participants aged 5 and 19 years. Data on fluid intelligence were assessed via a German version of the culture fair intelligence test (CFT), using CFT 1-R in children 8.5 years of age or younger (*n* = 62) or CFT 20-R in participants older than 8.5 years (*n* = 192). Multivariable linear regression models were used to investigate prospective associations between the lifestyle score and the fluid intelligence score.

**Results:**

Mean lifestyle score of all participants was 2.2 (0.7–4) points. A one-point increase in the lifestyle score was associated with a higher fluid intelligence score (4.8 points [0.3–7.3],* p* = 0.0343) for participants completing the CFT 20-R. Furthermore, each additional hour of sedentary behaviour was associated with a lower fluid intelligence score (− 3.0 points [− 5.7 to − 0.3],* p* = 0.0313). For younger participants (CFT 1-R), no association was found in any analysis (*p* > 0.05).

**Conclusion:**

A healthy lifestyle was positively associated with fluid intelligence, whereby sedentary behaviour itself seemed to play a prominent role.

**Supplementary Information:**

The online version contains supplementary material available at 10.1007/s00394-022-02921-z.

## Introduction

Accumulated cognitive reserve across the lifespan might play a role in the prevention of neurodegenerative diseases [1], which has turned into a major societal challenge implied by demographic change [2]. Intelligence, educational, and in later life occupational qualifications, as well as participation in intellectual and social leisure activities might contribute to the establishment of a reserve of skills [3, 4]. Some studies in adults have already shown a positive association between the lifestyle factors diet, physical activity, social or intercultural activities with cognitive function in later life [5–7], which might be mediated by cognitive reserve [5]. In addition, several studies looking at cognitive abilities across the lifespan associated higher cognitive abilities in childhood with reduced risk of dementia [8–10]. Moreover, a recent systematic review evaluated the evidence of childhood cognitive abilities and the risk for Alzheimer's dementia. Results showed that the risk for late onset of Alzheimer's dementia was higher in participants with lower intelligence during childhood than in participants with high cognitive abilities during childhood, predominantly in females [11]. These findings support the hypothesis of higher cognitive abilities in earlier life stages being beneficial to prevent later cognitive decline. Executive functions, which cover cognitive processes of working memory, inhibitory control, cognitive flexibility, planning, reasoning and problem solving, have an important role within cognitive abilities [12]. In addition, its development starts in childhood and adolescence, peaks in early adulthood and declines in later life [13].

Various single lifestyle factors in childhood and adolescence have been suggested to be associated with cognitive abilities [14–16]. For example, adherence to a healthy dietary pattern rich in fruits, vegetables, whole grains and fish and limited consumption of sugar-sweetened beverages, red and processed meat resulted in higher executive functions compared to less adherence to the pattern [14]. In addition, a recent summary of evidence of the World Health Organisation (WHO) supported the hypothesis that high levels of physical activity and low levels of sedentary behaviour during leisure time have a positive impact on cognitive abilities [15]. Furthermore, sleep behaviour has been associated with cognitive abilities in younger populations [16].

Recent studies investigated the associations between lifestyle patterns and academic achievement in children [17–19] or adolescents [20, 21]. Included lifestyle factors were diet and physical activity [17–21], sedentary behaviour or screen time [17, 18, 20, 21], sleeping habits [18, 20, 21], as well as cardio-respiratory fitness and body weight status [21]. Despite study results generally supporting the hypothesis of a positive association between a healthy lifestyle pattern and academic achievement, some inconsistency is left. Of note, several aspects such as the impact of being exposed to combined lifestyle factors compared to the influence of each individual lifestyle factor and sex differences need further exploration. Further, outcome measures might depict different aspects of cognitive abilities. For example, academic performance describes the extent to which a participant has achieved short- or long-term educational goals [22], while fluid intelligence on the contrary denotes a problem-solving ability based on nonverbal abstract thinking [23]. However, it is difficult to draw conclusions about fluid intelligence or other cognitive domains from studies investigating academic performance. Therefore, we aimed to investigate the association between lifestyle patterns in childhood and adolescence and subsequently measured fluid intelligence.

## Research design and methods

### Study design

The DOrtmund Nutritional and Anthropometric Longitudinally Designed (DONALD) study is an ongoing open cohort study, which was initiated in 1985. A detailed description of the study design has been reported elsewhere [24]. Briefly, repeated annual examinations of dietary intake, anthropometry, participants’ physical activity profile and sleep duration were conducted from the age of 3 months onwards. In addition, data on socio-economic parameters were updated occasionally. As part of an add-on module, data on fluid intelligence was collected once in 2017 and 2018 in a sample of 6–32-year-old participants. The DONALD study was conducted according to the Declaration of Helsinki and all investigations involving human subjects were approved by the Ethics Committee of the University of Bonn and were carried out with written informed consent of the study participant or parents.

### Study population

**Fig. 1:**
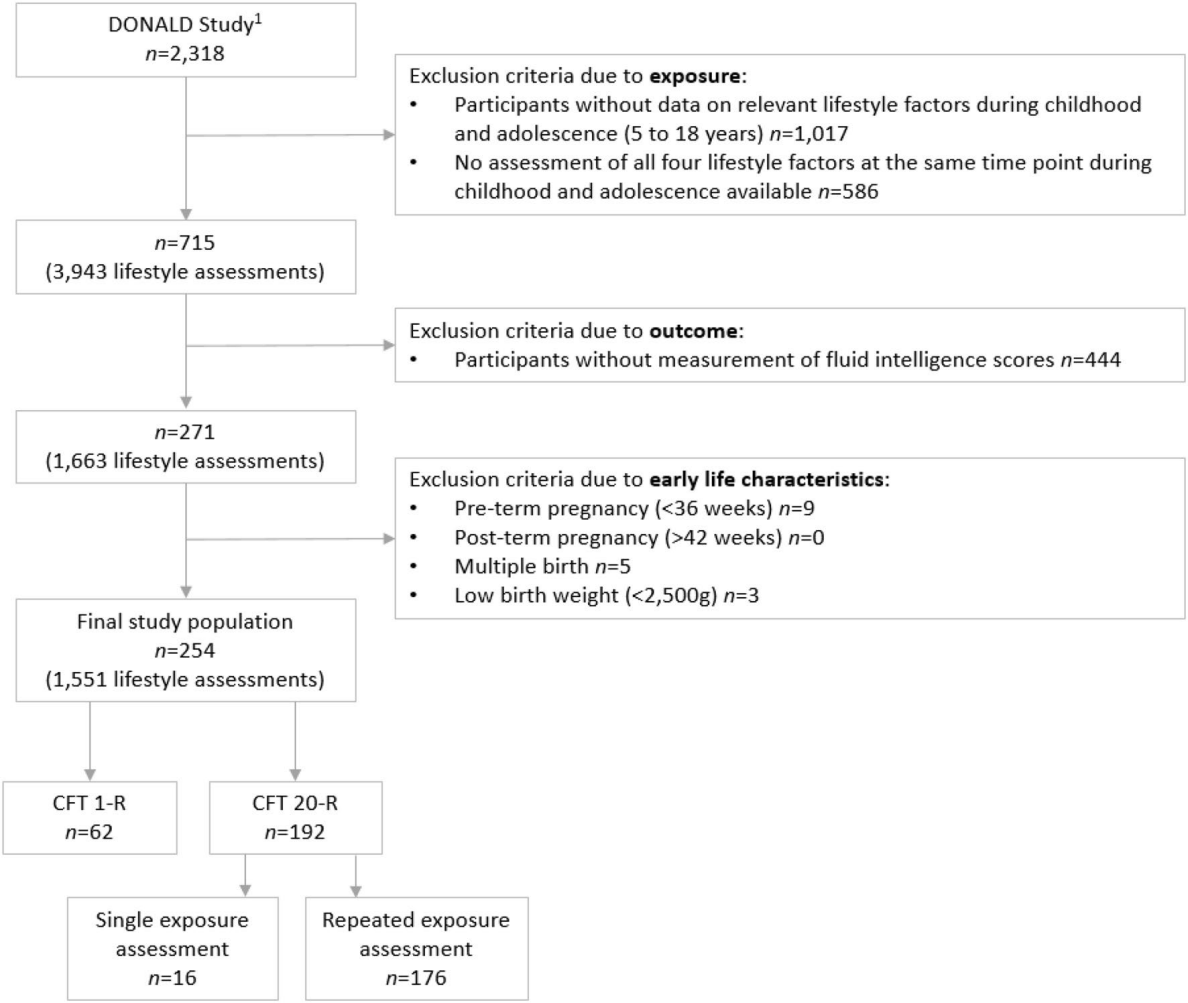
Flow diagram for participant data from the DONALD Study. *CFT* culture fair intelligence test. ^1^Participants were recruited between 1985 and September 2021

Participants of the DONALD study, who provided at least one measurement of all four lifestyle factors (diet, physical activity, sedentary behaviour and sleep duration) at the same age during childhood and adolescence (5–19 years) were included (*n* = 715). Of these, 271 participants took part in the add-on module “cognition”, where we assessed fluid intelligence using the culture fair intelligence test (CFT). Participants were not included if they were pre-term or post-term (< 36, or > 42 gestation week, respectively, *n* = 9), part of multiples (*n* = 5) or had a low birth weight (< 2500 g, *n* = 3). The final study population comprised in total 254 participants. Among these, 176 participants provided data on any lifestyle factor at least twice during childhood and adolescence (Fig. 1). S1 figure additionally shows a diagram depicting the repeated assessment of the four lifestyle factors during childhood and adolescence (exposure) and the subsequent measurement of the fluid intelligence score (outcome).

### Repeated assessment of lifestyle factors

Data on dietary intake were assessed using 3-day weighed dietary records (3dWR) from consecutive days [24]. Data quality regarding potential energy underreporting was tested by the Goldberg approach [25], using paediatric cut-offs for the ratio between reported total energy intake and estimated basal metabolic rate [26]. Participants who reported less energy intake than expected according to the Goldberg approach, have consciously or unconsciously written down less food items or quantities than they have eaten, or might have eaten less due to personal reasons. Duration of daily MVPA and sedentary behaviour (watching television and doing school homework) on weekdays and weekend days was assessed via questionnaires [27, 28]. Daily sleep duration was recorded via the question “How many hours per day do you sleep?”.

### Lifestyle score during childhood and adolescence (exposure)

The lifestyle score included four factors: dietary intake (fruits, vegetables, wholegrain, sugar-sweetened beverages, fish as well as red meat and sausages), duration of moderate-to-vigorous physical activity (MVPA), sedentary behaviour and sleep. We based it on a recently published hypothesis-based lifestyle score [29]. Scoring points were assigned when fulfilling pre-specified age-specific references for each factor at each age-specific time point. The age-specific scoring system is shown in Table 1. Further detailed information on portion size cut-offs for the considered food groups subdivided by age groups can be found in S1 Table. In our study population, healthy behaviours were scored 1 vs. 0 points for less favourable. Healthy behaviours were defined as (1) consumption of recommended servings per day in ≥ 3 food groups [30–32], (2) ≥ 60 min of MVPA per day [33], (3) sedentary time per day ≤ age-specific recommendations [34] and 4) being within the recommended range of hours of sleep per day [35, 36]. Points for all four lifestyle factors were summed up to a lifestyle score ranging from 0 to 4 points. To describe the lifestyle during childhood and adolescence, which in our study covered between 1 and 12 measurements per participant, the arithmetic mean of all available lifestyle scores per participant were calculated.

**Table 1:** Lifestyle factors, scoring categories, and references

Lifestyle factor and scoring criteria for age groups	Points	Recommendation
Overall score	0–4	
Dietary intake		
Consumption of < 3 food groups^a^/day	0	DGE [30], USDA [31], WHO [32]
Consumption of ≥ 3 food groups^a^/day	1	
MVPA		
< 60 min/day	0	WHO [33]
≥ 60 min/day	1	
Sedentary behaviour		
5 years: > 30 min/day	0	Graf et al. [34]
6–11 years: > 60 min/day		
12–19 years: > 120 min/day		
5 years: ≤ 30 min/day	1	
6–11 years: ≤ 60 min/day		
12–19 years: ≤ 120 min/day		
Sleep duration		
5 years: < 10 and > 13 h/day	0	AASM [35, 36]
6–12 years: < 9 and > 12 h/day		
13–17 years: < 8 and > 10 h/day		
18–19 years: < 7 and > 9 h/day		
5 years: 10–13 h/day	1	
6–12 years: 9–12 h/day		
13–17 years: 8–10 h/day		
18–19 years: 7–9 h/day		

*MVPA* moderate-to-vigorous physical activity, *DGE* German Nutrition Society, *USDA* United States Department of Agriculture, *WHO* World Health Organisation, *AASM* American Academy of Sleep Medicine

^a^Recommended servings were considered for the food groups fruits, vegetables, whole grains, sugar-sweetened beverages, fish and red meat/sausages

### Assessment of the fluid intelligence score (outcome)

Data on fluid intelligence were collected in 2017 and 2018 as part of an add-on module with the German version of the language-free and figure-based CFT [37, 38]. Two different tests types were used: CFT 1-R was used for participants less than 8.5 years of age and CFT 20-R was used for participants 8.5 years of age or older. Each test had a multiple-choice format and all items were sorted by increasing difficulty. The results from the age-specific CFTs were used to calculate a fluid intelligence score, which was used as outcome in our current study.

#### CFT 1-R

The interview-based paper-and-pencil test measures fluid intelligence with figural tasks continuation, classification and matrices (15 tasks each) and has been reported to take about 40 min to complete. Correct answers across all three subtests were added to a raw score and converted into an age-standardised fluid intelligence score. The CFT 1-R is known for its high retest-reliability with *r* = 0.95 [37].

#### CFT 20-R

The self-administered computer test with automatic evaluation has been reported to take about 1 h to complete. It contained two parts (56 and 45 items, respectively) with the following figural tasks: continuation of series, classifications, matrices, and topological conclusions. Correct answers across all four subtests were summed up and converted into an age-standardised fluid intelligence score. The retest-reliability of the CFT 20-R has been reported as high with *r* = 0.96 [38].

### Assessment of additional variables

Family and socioeconomic characteristics, such as parental education, employment, and smoking in the household were collected via interviews. Participants’ weight was measured according to a standard procedure, with participants dressed in underwear only and barefoot. Exclusive breastfeeding duration (weeks) was recorded via repeated parental interviews during the first year of life.

### Statistical analysis

All analyses were stratified according to CFT type given the fact that the two test variants addressed different age groups. Continuous variables are presented as median ± IQR and categorical variables as relative frequencies (%) unless otherwise noted. The longitudinal association between the lifestyle score and the fluid intelligence score was analysed using multivariable linear regression models with the lifestyle score as continuous variable. As the fluid intelligence score was normally distributed, no mathematical transformation was necessary. Potential confounders were included in the models if they significantly modified the predictor-outcome associations (change in ß-estimate ≥ 10%) [39]. Potential confounding variables considered were family and socioeconomic characteristics, a range of gestational, birth and early life parameters, participants’ weight and maternal overweight (BMI ≥ 25 kg/m^2^). Additionally, age at CFT and time difference between the mean lifestyle score assessment and CFT were also taken into account. A basic model was adjusted for age at CFT and sex. A multivariable adjusted model was additionally adjusted for parental education (< 12 vs. ≥ 12 years of education), smoking status in the household (never/current), exclusive breastfeeding for ≥ 4 month (yes/no) and participants weight (kg). Second, given that the role of sedentary behaviour while doing school homework as opposed to watching television might have different associations with respect to fluid intelligence, we replaced total sedentary behaviour with time spent watching TV, and alternatively with time spent doing school homework. Third, we calculated four modified lifestyle scores based on only three lifestyle factors instead of four, omitting one factor at a time. In this analysis, we additionally adjusted the multivariable models for the omitted lifestyle factor, respectively.

Additional sensitivity analyses in subsamples were performed: (1) participants who provided data on any lifestyle factor at least twice (*n* = 176), (2) participants with most reliable dietary data, i.e. excluding those at risk of potential underreporting of energy (*n* = 186) and (3) participants who had a time difference between exposure and outcome assessment < 10 years (*n* = 152). For the current sample, a *post-hoc* power analysis was conducted using G*Power 3.1 [40]. Our power analysis showed that this study including in total 254 participants, considering an *α* of 0.05, is adequately powered (80%) to observe even small effect sizes up to a Cohen’s *f*^2^ of 0.03. All statistical analyses were run using SAS (Version 9.4; Cary, NC, USA). Statistical significance was defined as a *P* value < 0.05.

## Results

Descriptive characteristics of all 254 participants stratified by test type are shown in Table 2. Mean age at cognitive testing was 6.9 (5.9–8.3) years for CFT 1-R tests and 18.1 (8.6–31.8) years for CFT 20-R tests, whereby participants completing CFT 1-R had a mean fluid intelligence score of 106 (78–133) points and participants of the CFT 20-R group had a mean fluid intelligence score of 111 (72–152) points. On average, participants provided data on lifestyle factors 5.4 times during the age of 5–19 years, ranging from 1 to 12 measurements. Around 85% of all participants provided at least two complete measurements of all lifestyle factors. The lifestyle score for all study participants was 2.2 (0.7–4) points, whereby younger participants (CFT 1-R) had a lifestyle score of 2.7 (1–4) points and older participants (CFT 20-R) had a lifestyle score of 2.1 (0.7–4) points. Moreover, participants of the CFT 1-R group had a quite homogeneous high lifestyle with few differences in the overall lifestyle score (≥ 2.5 points: 66.1% of the participants), showing a lack of variance in the individual lifestyle factors. In addition, more than 98% of these children followed age-specific recommendations for sleep duration and nearly half of them were able to achieve the recommendations for MVPA (Table 3). In the CFT 20-R group, a lifestyle score ≥ 2.5 points was achieved by 25.5% of participants, and was thus more heterogeneous.

**Table 2:** Participant characteristics^a^ according to the CFT type

	CFT 1-R (*n* = 62)	CFT 20-R (*n* = 192)
Age at cognitive testing (years)^b^	6.9 (5.9–8.3)	18.1 (8.6–31.8)
Mean age at lifestyle assessment (years)^b^	6.1 (5.0–7.1)	11.6 (5.0–18.9)
Male participants (%)	56.5	49.0
Lifestyle score^b^	2.7 (1–4)	2.1 (0.7–4)
Fluid intelligence score^b^	106 (78–133)	111 (72–152)
Parental education (≥ 12 years, %)	93.6	75.0
Smoking in the household (yes, %)	9.7	15.1
Exclusive breastfeeding (≥ 4 month, %)	72.6	72.4
Body weight (kg)	22.1 (4.0)	41.8 (25.6)
≥ 2 lifestyle assessments (%)	66.1	91.7
Lifestyle factors during childhood and adolescence		
Fruits (g/day)	279.3 (229.6)	266.3 (215.8)
Vegetables (g/day)	92.7 (99.7)	108.0 (70.8)
Whole grain (g/day)	31.1 (52.1)	33.3 (48.3)
Sugar-sweetened beverages (g/day)	15.1 (77.8)	126.0 (204.4)
Fish (g/day)	1.9 (23.6)	10.5 (17.7)
Red meat and sausages (g/day)	52.3 (42.5)	66.8 (47.8)
MVPA (min/day)	74.9 (39.3)	67.1 (38.0)
Sedentary behaviour (min/day)	54.3 (34.3)	138.1 (90.0)
Watching television (min/day)	43.9 (30.0)	79.9 (58.6)
Doing school homework (min/day)	7.1 (14.3)	43.3 (40.9)
Sleep duration (h/day)	10.5 (0.8)	9.2 (1.4)

*CFT* culture fair intelligence test, *MVPA* moderate-to-vigorous physical activity

^a^Data shown as median (interquartile range) or relative frequency (%)

^b^Mean (min–max)

**Table 3:** Proportion of participants fulfilling the reference (in %)

	CFT 1-R (*n* = 62)	CFT 20-R (*n* = 192)
Healthy diet (≥ 3 food groups/day)	40.3	12.5
MVPA (≥ 60 min/day)	53.2	14.1
Sedentary behaviour (≤ age-specific reference/day)	40.3	6.3
Sleep duration (within age-specific reference/day)	98.4	45.3

*MVPA* moderate-to-vigorous physical activity

**Fig. 2:**
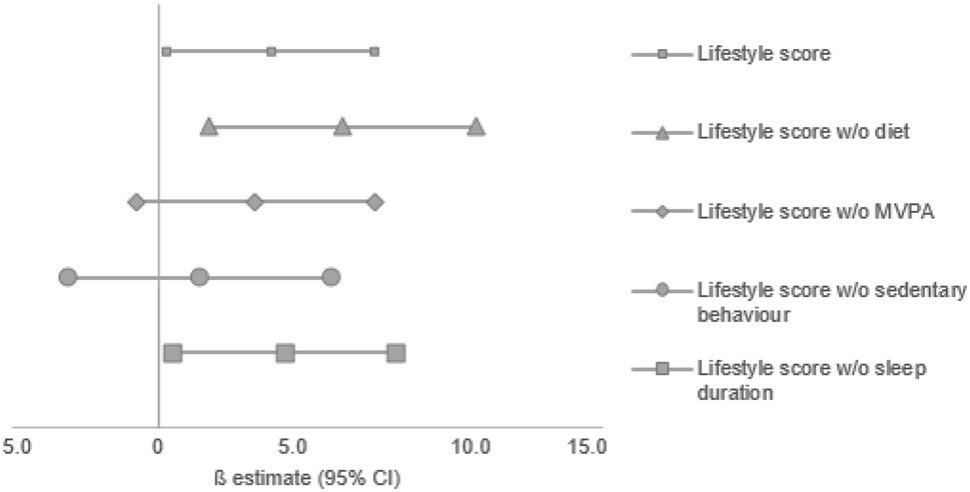
Change in fluid intelligence score per 1-point increase in the lifestyle score and modified versions (CFT 20-R participants only). Associations were analysed using multiple linear regression. Analysis were adjusted for age, sex, parental education, smoking in the household, exclusive breastfeeding, body weight, and the omitted lifestyle factor. *MVPA* moderate-to-vigorous physical activity, *CFT* culture fair intelligence test

For participants in the CFT 1-R group no association between the lifestyle score and fluid intelligence score was observed in any of our analyses (Table 4 and supplementary material). In the CFT 20-R group, a higher lifestyle score was associated with a higher fluid intelligence score after adjustment for potential confounders (multivariate adjusted model: 3.8 [0.3–7.3] points, *p* = 0.0343). When separately using watching television or doing school homework as a proxy for sedentary behaviour instead of the combination, associations were no longer significant (S2 Table). Modified lifestyle scores without either diet and sleep duration remained positively associated with the fluid intelligence score (6.2 [1.7–10.7] points, *p* = 0.0077 and 4.2 [0.5–8.0] points, *p* = 0.0280, respectively) (Fig. 2). After excluding factors of participants activity profile (MVPA or sedentary behaviour), no significant associations remained (*p* > 0.05). Moreover, we have analysed MVPA and sedentary behaviour as single factors to determine the specific association with fluid intelligence scores: each additional hour of sedentary behaviour was inversely associated with the fluid intelligence score (− 3.0 [− 5.7 to − 0.3] points, *p* = 0.0314). Each additional hour of watching TV, doing school homework or MVPA was not associated with the fluid intelligence score (*p* > 0.05, data not shown).

**Table 4:** Association between lifestyle score and fluid intelligence

	CFT 1-R (*n* = 62)		CFT 20-R (*n* = 192)	
	*ß* (95% CI)	*P-*value	*ß* (95% CI)	*P-*value
Basic model	− 3.3 (− 7.8 to 1.2)	0.14	4.0 (0.6 to 7.5)	0.0208
Multivariate adjusted model	− 4.1 (− 8.7 to 0.6)	0.09	3.8 (0.3 to 7.3)	0.0343

Associations were analysed using multiple linear regression. Basic model adjusted for age and sex, multivariate adjusted model: basic model + additionally adjusted for parental education, smoking in the household, exclusive breastfeeding and body weight

*CFT* culture fair intelligence test

The results of our sensitivity analyses with participants who provided data on any lifestyle factor at least twice (*n* = 176) showed comparable results (data not shown). When analysing participants who provided more correct than potentially underreported 3dWR (*n* = 186) or participants with a time difference between exposure and outcome assessment < 10 years (*n* = 152), no significant association remained (data not shown).

## Discussion

In our study, adherence to a healthy lifestyle was positively associated with a fluid intelligence score in participants 8.5 years of age or older. Aspects of physical activity including duration of sedentary behaviour seemed to play a specific role in the association. An in-depth analysis has shown that each additional hour of sedentary time was negatively associated with fluid intelligence. However, our models were sensitive to exclusions, showing the need for further replication of our findings. No significant association for children aged 6–8.5 years was observed.

To our knowledge, no previous study has investigated the longitudinal association between a combination of lifestyle factors measured repeatedly during childhood and adolescence and subsequently measured fluid intelligence. Five studies have cross-sectionally investigated associations between independent and combined lifestyle factors with academic performance in school-aged children [17–21], in which the authors have combined between two and six individual lifestyle factors. In general, the authors used the same four lifestyle factors as we did [17–21], and one study additionally used cardio-respiratory fitness and body weight status [21]. High adherence to combined lifestyle recommendations was associated with meeting the goals for mathematics, reading or writing [17–21]. Although academic performance is not fully comparable with fluid intelligence, our findings point into the same direction.

For the participants of the CFT 1-R, no statistically significant results could be identified. We hypothesise various reasons. First, the total sample size of this study is relatively modest with *n* = 254. Of these, 62 participants filled in the CFT 1-R. This sample size allows observing small medium effect sizes (Cohen’s *f*^2^ ≥ 0.13), considering an alpha-level of 0.05 and a power 80%. Hence, our study might have been too small to observe significant associations. Second, due to a lack of variance in individual lifestyle factors, participants of the CFT 1-R group had a quite homogeneous high lifestyle score (≥ 2 points: 91.5%). In addition, almost 97% of these children adhered to the age-specific recommendations for sleep duration, and almost half of them were able to achieve the dietary recommendations (Table 3). Third, the variation in lifestyle score also was small due to the short follow-up time of the young participants. On average, participants had two lifestyle assessments, whereas participants in the CFT 20-R group had more than six lifestyle assessments.

Different subcategories of general intelligence, such as crystalline or fluid intelligence, as well as auditory or visual perception or processing speed [41] have generally been assessed with different types of intelligence test [42, 43]. As fluid intelligence represents problem-solving capacity and is considered independent of learning, experience as well as education [23], we opted for the assessment of this dimension in our study—also meeting a limited time space for this module in our study design. We decided to assess fluid intelligence with the CFT, a nonverbal test that avoids cultural and linguistic biases and focuses on reasoning. In addition, the CFT examines age-standardised values of fluid intelligence and, thus is usable for populations with wide age ranges [37, 38], such as in our study population ranging from 6 to 32 years.

A growing body of studies exists, analysing individual associations between healthy dietary patterns [14, 44, 45], physical activity [15, 45–47], sedentary behaviour [15, 48, 49] or sleep behaviour [16, 50, 51] in children and adolescents with cognitive abilities. Consistent evidence was shown for a positive association between a healthy dietary pattern [14, 44, 45] as well as an active lifestyle [15, 45–47, 49] and cognitive abilities. Instead of analysing the whole diet, we decided to focus on the dietary factors fruits and vegetables [52–54], whole grain [55], sugar-sweetened beverages [54, 56], fish [57, 58] and red meat [44, 59], as these factors seems to play a major role in the context of different cognitive abilities. We also decided to include MVPA [15, 45] and sedentary behaviour [15, 47] in the lifestyle score, as these measures appear to have been widely used in the literature to determine the relationship between participants’ activity profile and cognitive abilities. Moreover, we included sleep duration, as recent systematic reviews and meta-analyses have shown the important, often not fully understood and potentially underestimated role of sleep in cognitive processes [16, 50, 51].

To account for a potentially dominating impact of one specific lifestyle factor, we created four modified lifestyle scores, each based on three lifestyle factors, and additionally adjusted the multivariate adjusted model of our regression analysis for the factor left out in the score. Our findings indicate a prominent role for aspects of physical activity, specifically sedentary behaviour in the relationship. Furthermore, our single factor analysis of sedentary behaviour has shown its specific importance in the context of cognitive abilities. Consistent with our findings are results from studies that have examined the association between lifestyle and academic achievement in both combined and single factor analyses [17–21]. In addition to overall lifestyle, meeting recommendations of the single factor of screen time as a proxy for sedentary behaviour was associated with academic achievement [18] and academic performance [21]. Moreover, systematic reviews showed promising results supporting the suggestion that predominantly low sedentary behaviour has a positive impact on cognitive function and brain structure [48, 49].

Studies in adults suggest that a healthy and active lifestyle is associated with cognitive reserve and, thus, favour the maintenance of cognitive functions in old age. Health-promoting diets [5] and physical activity [5–7], as well as education [7, 60] and engaging in cognitive or social activities [5–7, 61, 62] enhance the cognitive reserve. Evidence from these studies implies that healthy lifestyle choices might help protect against clinical manifestations of dementia. In addition to the factors diet and physical activity, education, employment, social and cognitive activities seem to be important. Some of them (participants’ education, social and cognitive activities) were not assessed in the DONALD study. Therefore, it was not possible to include these in our lifestyle score. Nevertheless, we adjusted for parental education to account for the social economic status, which has been related to fluid intelligence in the past [7, 60]. Indeed, there was a positive association between higher parental education (< 12 years vs. ≥ 12 years) and the participant’s fluid intelligence score (ß-estimate for the CFT 20-R group: 5.0 points, *p* = 0.0454).

According to a WHO report, sedentary behaviour while reading and doing school homework is associated with higher academic achievement, suggesting that there are differences according to the type of activity [15]. When we divided the sedentary behaviour variable into two independent variables watching TV and doing school homework, no significant association between lifestyle score and the fluid intelligence score was found (*p* > 0.05). In line with the WHO, we speculate that the type of mental activity performed during sedentary time might modulate the effect of sedentary behaviour on fluid intelligence. Non-screen-based activities, such as school homework or reading, were positively associated with academic performance [49, 63], supported by several recent observational studies [64, 65]. The impact of screen-based activities on cognitive dimensions seems to be more complex and dependent on the type of screen (television, computer, video games or phone) and content (educational or recreational program). While general screen time was negatively associated with cognition [48], there were indications that educational television might be positively associated [66]. However, we were unable to divide our data regarding watching entertainment television or educational programmes, and anyway worked with a study population most certainly too small for more stratified analyses.

### Limitations and strength

However, our study has several limitations. The choice of lifestyle factors to be included in our lifestyle score was somewhat arbitrary and challenging [67]; however, we included a number of lifestyle factors (diet, physical activity, sedentary behaviour, sleep duration) with the intention to be as consistent as possible with the existing literature on lifestyle patterns in the field of cognitive abilities in young age. In addition, we have recently shown that the developed lifestyle score is an appropriate description of the adolescent lifestyle [29] and we further do not expect the lifestyle score to misrepresent actual lifestyle, due to its hypothesis- and recommendation-based manner. Nonetheless, additional factors such as daily screen time as a sum of television, computer and smartphone use, or social contacts might have been of interest, as discussed above. Standardised questionnaires on the self-reported sedentary behaviour and sleep duration have not been validated. However, the questionnaire for sedentary behaviour has been used in a nationwide cohort [28]. Furthermore, our participants are characterised by a high socioeconomic status compared to the general German population [24], limiting the generalisability of our results. Confounding by potentially unmeasured covariates, such as family history of cognitive decline, remains possible.

The analysis is characterised by several strengths, including the longitudinal design, which allowed us to create a lifestyle score on repeated measurements. Self-reported food intake data were collected using 3dWR, a generally very detailed and valid instrument to assess quantitative food intake. The latter was important, since the score was created based on recommendations for absolute intake. When analysing participants with a low risk of potential energy underreporting the significant association disappeared (*p* = 0.0547), probably due to the fact that the already small study population has become even smaller (*n* = 186). However, diet is only one of four lifestyle factors used and little is known about the quality of the measurement and the evaluation of the individual factors. To collect data on MVPA, we use a validated questionnaire [27]. However, due to the self-reported manner and the fact that questionnaires often tend to overestimate activity, it is prone to measurement errors or other biases [68]. Objective and thus more reliable measures of physical activity were not used in our study at the time of data collection. Lastly, we used reference-based cut-offs [30–36] instead of population-specific cut-offs to ensure comparability with existing and future literature. Furthermore, we used cut-offs according to age-specific recommendations for MVPA, sedentary behaviour and sleep duration [30–36], as well as age-specific recommendations for portion sizes (S1 Table). With this approach, we were able to consider age-specific recommendations throughout childhood and adolescence within and between participants.

## Conclusion

Adherence to a combined lifestyle score including diet, physical activity, sedentary behaviour and sleep duration in childhood and adolescence was positively associated with subsequently measured fluid intelligence scores in study participants aged 8.5–32 years. In this context, physical activity, specifically sedentary behaviour, was of importance. As fluid intelligence is part of a person’s cognitive reserve, a comprehensive understanding of its relationship with lifestyle factors over the lifespan is needed. As our sensitivity analyses showed limited robustness of our findings, further research into the topic is warranted.

## Supplementary Information

Below is the link to the electronic supplementary material.

Supplementary file1 (DOCX 16 kb)
