# Supplementary material for: A lifestyle score in childhood and adolescence was positively associated with subsequently measured fluid intelligence in the DONALD cohort study
Source: Eur J Nutr. 2022 Jun 15;61(7):3719–29. doi: 10.1007/s00394-022-02921-z (PMC9464141; doi:10.1007/s00394-022-02921-z)
Supplement: Supplementary file 1 — Supplementary file1 (DOCX 16 kb) [file 394_2022_2921_MOESM1_ESM.docx]

**Supplementary material for “A lifestyle score in childhood and adolescence was positively associated with subsequently measured fluid intelligence in the DONALD cohort study”**

**S1 Table:** Reference values for serving sizes (g/d) by age

| **Dietary factor** | **5 yrs** | **6 yrs** | **7 yrs** | **8 yrs** | **9 yrs** | **10 yrs** | **11 yrs** | **12 yrs** | **13 yrs** | **14 yrs** | **15 yrs** | **16 yrs** | **17 yrs** | **18 yrs** |
| --- | --- | --- | --- | --- | --- | --- | --- | --- | --- | --- | --- | --- | --- | --- |
| **Fruits (g)** |  |  |  |  |  |  |  |  |  |  |  |  |  |  |
| fresh | 82.0 | 87.5 | 87.0 | 95.0 | 96.0 | 100.0 | 102.0 | 104.0 | 110.0 | 118.0 | 122.0 | 121.0 | 122.0 | 122.0 |
| dried | 9.7 | 10.0 | 12.5 | 10.0 | 10.9 | 10.0 | 10.0 | 10.0 | 10.0 | 14.7 | 11.8 | 12.0 | 16.5 | 18.9 |
| juice | 92.0 | 100.0 | 106.0 | 112.0 | 120.0 | 126.0 | 134.0 | 147.6 | 156.0 | 156.0 | 175.0 | 173.0 | 175.0 | 170.0 |
| **Vegetables (g)** |  |  |  |  |  |  |  |  |  |  |  |  |  |  |
| fresh | 48.0 | 50.0 | 55.7 | 57.6 | 59.0 | 61.8 | 66.3 | 70.5 | 73.6 | 78.3 | 77.0 | 81.1 | 82.0 | 88.0 |
| salad | 15.4 | 25.0 | 25.6 | 25.0 | 25.2 | 28.9 | 30.0 | 26.4 | 32.3 | 32.8 | 34.6 | 30.0 | 30.0 | 30.7 |
| legumes | 36.0 | 45.6 | 46.0 | 57.0 | 55.9 | 51.1 | 63.8 | 65.1 | 61.2 | 70.2 | 73.9 | 77.5 | 74.1 | 74.8 |
| juice | 75.0 | 111.5 | 80.0 | 121.8 | 120.0 | 138.0 | 150.0 | 165.0 | 124.0 | 130.0 | 150.0 | 170.0 | 323.2 | 77.4 |
| **Wholegrain products (g)** | 38.0 | 42.0 | 44.0 | 47.0 | 48.0 | 50.0 | 52.3 | 54.0 | 55.0 | 61.0 | 63.0 | 64.8 | 68.0 | 72.0 |
| **Sugar-sweetened beverages (g)** | 165.0 | 188.0 | 200.0 | 200.0 | 200.0 | 200.0 | 208.0 | 225.0 | 250.0 | 250.0 | 280.0 | 289.5 | 283.0 | 292.0 |
| **Fish (g)** | 55.0 | 61.9 | 62.0 | 71.0 | 66.2 | 72.0 | 70.2 | 85.0 | 82.2 | 93.1 | 88.0 | 79.8 | 88.0 | 90.6 |
| **Red meat and sausages (g)** |  |  |  |  |  |  |  |  |  |  |  |  |  |  |
| red meat | 42.3 | 48.0 | 50.0 | 54.0 | 59.6 | 60.0 | 64.6 | 70.0 | 78.5 | 83.2 | 80.0 | 86.1 | 88.3 | 92.2 |
| sausages | 18.0 | 18.3 | 19.0 | 20.0 | 20.0 | 20.0 | 21.0 | 21.8 | 22.0 | 21.0 | 23.0 | 25.0 | 24.9 | 25.0 |

**S2 Table:** Association between adherence to lifestyle factor recommendations and fluid intelligence with modifications in the lifestyle factor sedentary behaviour

|  | CFT 1-R (n=62) | | CFT 20-R (n=192) | |
| --- | --- | --- | --- | --- |
|  | ß (95% CI) | *P-*value | ß (95% CI) | *P-*value |
| Lifestyle score with watching television^1^ | -4.6 (-9.2 to 0.1) | 0.06 | 2.8 (-0.7 to 6.2) | 0.11 |
| Lifestyle score with doing homework^2^ | -5.5 (-11.5 to 0.5) | 0.07 | 2.9 (-1.2 to 7.0) | 0.17 |

CFT: culture fair intelligence. Associations were analysed using multiple linear regression. Analysis were adjusted for age, sex, parental education, smoking in the household, exclusive breastfeeding and body weight. ^1^Lifestyle score consists of the factors diet, moderate-to-vigorous physical activity, time spent watching television and sleep duration, ^2^Lifestyle score consists of the factors diet, moderate-to-vigorous physical activity, time spent doing homework and sleep duration.
